# Identification of novel molecular markers of mastitis caused by *Staphylococcus aureus* using gene expression profiling in two consecutive generations of Chinese Holstein dairy cattle

**DOI:** 10.1186/s40104-020-00494-7

**Published:** 2020-09-28

**Authors:** Di Wang, Lei Liu, Serafino M. A. Augustino, Tao Duan, Thomas J. Hall, David E. MacHugh, Jinhuan Dou, Yi Zhang, Yachun Wang, Ying Yu

**Affiliations:** 1 Key Laboratory of Animal Genetics, Breeding and Reproduction, Ministry of Agriculture & National Engineering Laboratory for Animal Breeding, College of Animal Science and Technology, China Agricultural University, Beijing, 100193 China; 2 UCD School of Agriculture and Food Science, University College Dublin, Dublin, D04 V1W8 Ireland; 3 Shenzhen Branch, Guangdong Laboratory for Lingnan Modern Agriculture, Genome Analysis Laboratory of the Ministry of Agriculture, Agricultural Genomics Institute at Shenzhen, Chinese Academy of Agricultural Sciences, Shenzhen, 518120 China; 4 Department of Animal Nutrition and Feed Science, College of Animal Science and Technology, China Agricultural University, Beijing, 100193 China; 5 UCD Conway Institute of Biomolecular and Biomedical Research, University College Dublin, Dublin, D04 V1W8 Ireland

**Keywords:** Dairy cow, Disease resistance, Mastitis, Peripheral blood leukocyte, *Staphylococcus aureus*, Transcriptome, Two generations

## Abstract

**Background:**

Mastitis in dairy cows caused by *Staphylococcus aureus* is a major problem hindering economic growth in dairy farms worldwide. It is difficult to prevent or eliminate due to its asymptomatic nature and long persistence of infection. Although transcriptomic responses of bovine mammary gland cells to pathogens that cause mastitis have been studied, the common responses of peripheral blood leukocytes to *S. aureus* infection across two consecutive generations of dairy cattle have not been investigated.

**Methods:**

In the current study, RNA-Seq was used to profile the transcriptomes of peripheral blood leukocytes sampled from *S. aureus*-infected mothers and their *S. aureus*-infected daughters, and also healthy non-infected mothers and their healthy daughters. Differential gene expression was evaluated as follows: 1) *S. aureus*-infected cows versus healthy non-infected cows (S vs. H, which include all the mothers and daughters), 2) *S. aureus*-infected mothers versus healthy non-infected mothers (SM vs. HM), and 3) *S. aureus*-infected daughters versus healthy non-infected daughters (SMD vs. HMD).

**Results:**

Analysis of all identified expressed genes in the four groups (SM, SMD, HM, and HMD) showed that *EPOR*, *IL9*, *IFNL3*, *CCL26*, *IL26* were exclusively expressed in both the HM and HMD groups, and that they were significantly (*P* <  0.05) enriched for the cytokine-cytokine receptor interaction pathway. A total of 17, 13 and 10 differentially expressed genes (DEGs) (FDR *P*_adj._ < 0.1 and |FC| > 1.2) were detected in the three comparisons, respectively. DEGs with *P* <  0.05 and |FC| > 2 were used for functional enrichment analyses. For the S vs. H comparison, DEGs detected included *CCL20*, *IL13* and *MMP3*, which are associated with the IL-17 signaling pathway. In the SM vs. HM and SMD vs. HMD comparisons, five (*BLA-DQB*, *C1R*, *C2*, *FCGR1A*, and *KRT10*) and six (*BLA-DQB*, *C3AR1*, *CFI*, *FCAR*, *FCGR3A*, and *LOC10498484*) genes, respectively, were involved in the *S. aureus* infection pathway.

**Conclusions:**

Our study provides insights into the transcriptomic responses of bovine peripheral blood leukocytes across two generations of cattle naturally infected with *S. aureus*. The genes highlighted in this study could serve as expression biomarkers for mastitis and may also contain sequence variation that can be used for genetic improvement of dairy cattle for resilience to mastitis.

## Background

Bovine mastitis is an inflammation-driven disease of the mammary gland in cows. It normally occurs in response to infection by one of a number of pathogenic microorganisms including *Escherichia coli* and *Staphylococcus aureus* [1, 2]. It is widely recognized that the high incidence of bovine mastitis, coupled with associated animal welfare problems and the use of antimicrobials, means that this disease is one of the major challenges facing the dairy industry in the twenty-first century [3]. *S. aureus* is an important type of Gram-positive bacteria and is defined as a “contagious pathogen” [4]. *S. aureus* can adapt to the mammary gland environment and establish subclinical infections [1, 5]. The symptoms of *S. aureus* mastitis are usually less severe than mastitis caused by infection with *E. coli*, and may even be asymptomatic; however, the infection can persist for long periods and can also exhibit marked resistance to antibiotics [6]. *S. aureus* typically spreads among cattle within dairy herds during the process of milking [7], making prevention, control and elimination particularly difficult. Therefore, bovine mastitis caused by this bacterial pathogen represents a substantial economic burden to dairy production and also poses a significant public health risk through milk consumption.

Global functional genomics technologies, such as RNA sequencing (RNA-Seq), which is enabled by high-throughput sequencing (HTS), are ideally suited for investigating the complex host-pathogen interaction underlying mastitis disease caused by *S. aureus* infection and may also provide relevant data for elucidating the molecular mechanisms associated with resilience to mastitis disease. RNA-Seq enables expression quantification analysis of thousands of genes simultaneously [6, 8], thereby facilitating identification of individual genes that exhibit the largest expression changes in response to a biological perturbation such as infection by a bacterial pathogen. Several transcriptomics studies have investigated the bovine mammary gland tissue response to *S. aureus* [9] and *E. coli* [10], and also the hepatic tissue response to *E. coli* infection and lipopolysaccharide (LPS) challenge [11, 12]. However, to-date, no studies have been carried out on the peripheral blood transcriptome for *S. aureus* infection in cows and their offspring simultaneously.

In the current study, using RNA-Seq, we characterized the transcriptomes of peripheral blood leukocyte (PBL) samples collected from *S. aureus*-positive mother-daughter pairs and *S. aureus*-negative control mother-daughter pairs. The main objective of this work was to identify candidate blood-based transcriptional biomarkers for bovine mastitis caused by *S. aureus* and to provide new insights into host-pathogen interaction and the genetics of disease resistance.

## Methods

### Animal selection and sampling

All procedures for collection of animal blood and milk samples were approved by the Animal Welfare Committee of China Agricultural University, Beijing, China. All experiments were conducted according to the regulations and guidelines established by this committee (permit number: DK996).

**Fig. 1:**
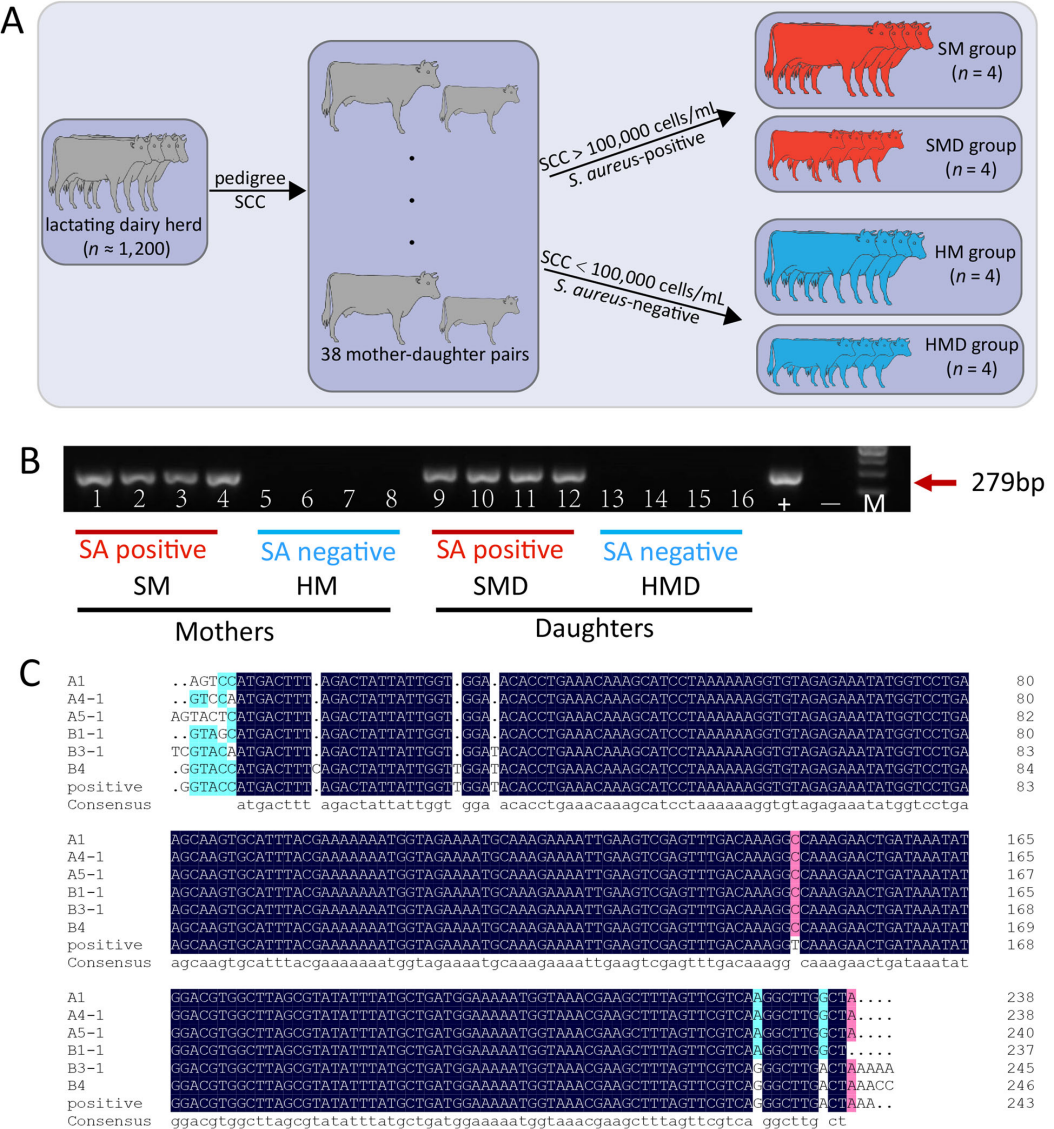
Identification of *S. aureus* isolated from milk samples from Holstein cows. **a** Workflow of sample selection. A total of 38 mother-daughter pairs were selected from 1,200 lactating dairy cattle according to pedigree and SCC records. After *S. aureus* identification from milk samples, four *S. aureus*-positive and four *S. aureus*-negative pairs were used for subsequent studies. **b** Specific PCR and electrophoresis map of the *S. aureus* thermonuclease (*nuc*) gene (279 bp). SA: abbreviation of *S. aureus*. +: *S. aureus*-positive control; −: *S. aureus*-negative control. SM: *S. aureus* mastitis mother; HM: healthy mother; SMD: *S. aureus* mastitis daughter; HMD: healthy daughter. c Sequence alignment of partial *nuc* gene. The upper lines were amplified from samples collected from the cows’ milk; the lower line is *nuc* sequence amplified from one of the *S. aureus*-positive samples. Identity = 95.68%

In the present study, a lactating dairy cow and her lactating mother were defined as a mother-daughter pair. A total of 38 mother-daughter pairs were selected based on pedigree information and somatic cell counts (SCC) from a lactating herd of Holstein cows (*n* ≈ 1,200) at a dairy farm near Beijing, China. The SCC data for three consecutive months were measured and recorded. The Dairy herd improvement (DHI) records were provided by the Dairy Data Centre of China (www.holstein.org.cn). For the present study, milk SCC values of less than 100,000 cells/mL from both mothers and daughters were considered to be healthy [13]. Conversely, SCC values larger than 100,000 cells/mL were considered for diagnosis of subclinical mastitis. Detailed information on cow selection is provided in Fig. 1a and Table 1.

**Table 1:** Basic information and bacterial culture of the 16 Chinese Holstein milk samples

Sample	Age	Days of lactation	SCC × 1000/mL			SCS			MY			FP			PP			Bacterium
			−3 months	−2 months	− 1 month	−3 months	−2 months	−1 month	−3 months	−2 months	−1 month	−3 months	−2 months	−1 month	−3 months	−2 months	−1 month	
SM1	8	482	561.00	911.00	1071.00	5.49	6.19	6.42	28.80	28.60	26.80	3.93	4.06	4.28	3.63	3.75	3.65	*S. aureus*
SM2	5	266	972.00	4641.00	4289.00	6.28	8.54	8.42	31.00	42.60	34.40	3.79	2.75	3.59	3.49	2.65	3.02	*S. aureus*
SM3	8	529	382.00	1076.00	852.00	4.93	6.43	6.09	36.70	37.50	35.80	2.14	3.97	4.10	3.38	2.98	2.91	*S. aureus*
SM4	5	291	155.00	585.00	241.00	3.63	5.55	4.27	31.30	31.30	24.60	2.10	3.81	3.52	3.42	3.29	3.30	*S. aureus*
Mean ± SD			517.5 ± 149.63	1803.25 ± 823.94	1613.25 ± 787.25	5.08 ± 0.48	6.67 ± 0.56	6.30 ± 0.74	31.95 ± 1.45	35 ± 2.72	30.4 ± 2.39	2.99 ± 0.44	3.65 ± 0.26	3.87 ± 0.16	3.48 ± 0.05	3.17 ± 0.20	3.22 ± 0.14	
SMD1	6	171	207.00	596.00	2249.00	4.05	5.58	7.49	58.00	50.90	41.60	3.67	3.46	3.51	2.96	3.11	3.24	*S. aureus*
SMD2	3	299	116.00	121.00	202.00	3.21	3.28	4.01	42.70	37.70	35.60	3.84	3.60	4.15	3.03	3.05	3.13	*S. aureus*
SMD3	3	125	283.00	102.00	64.00	2.99	1.49	2.96	25.70	25.50	24.70	2.76	3.26	2.85	3.31	3.06	3.32	*S. aureus*
SMD4	2	33	99.00	113.00	145.00	2.99	3.18	3.54	31.30	38.60	37.70	2.89	4.95	1.73	2.83	2.86	2.88	*S. aureus*
Mean ± SD			176.25 ± 37.03	233 ± 104.84	665 ± 457.92	3.31 ± 0.22	3.38 ± 0.73	4.50 ± 0.88	39.43 ± 6.18	38.18 ± 4.49	34.9 ± 3.14	3.29 ± 0.24	3.82 ± 0.33	3.06 ± 0.45	3.03 ± 0.088	3.02 ± 0.05	3.14 ± 0.08	
HM1	7	258	101.00	155.00	73.00	3.01	3.63	2.55	51.80	47.40	38.00	2.78	3.02	2.88	3.06	2.97	2.92	–
HM2	6	222	28.00	37.00	57.00	1.16	1.57	2.19	38.10	35.30	30.10	3.82	4.40	3.89	3.12	3.17	3.13	–
HM3	5	173	76.00	23.00	39.00	2.60	0.88	1.64	58.30	58.80	56.00	3.78	3.81	3.46	3.34	3.22	3.23	–
HM4	5	244	153.00	34.00	44.00	3.61	1.44	1.82	42.10	40.80	38.20	4.69	4.31	3.95	3.88	3.25	3.12	–
Mean ± SD			89.5 ± 22.54	62.25 ± 26.90	53.25 ± 6.58	2.60 ± 0.45	1.88 ± 0.52	2.05 ± 0.17	47.58 ± 3.97	45.58 ± 4.38	40.58 ± 4.74	3.77 ± 0.34	3.89 ± 0.27	3.55 ± 0.21	3.35 ± 0.16	3.15 ± 0.05	3.1 ± 0.06	
HMD1	3	145	22.00	19.00	27.00	0.82	0.60	1.11	35.70	40.30	38.80	3.91	3.66	3.53	3.01	3.18	3.08	–
HMD2	4	212	22.00	32.00	514.00	0.82	1.36	5.36	52.00	46.30	25.10	3.42	2.57	5.11	3.27	3.32	3.46	–
HMD3	3	271	60.00	20.00	61.00	2.26	0.68	2.29	42.90	42.70	35.80	3.31	3.30	2.60	3.17	3.14	3.18	–
HMD4	3	250	30.00	61.00	47.00	1.26	2.29	1.91	37.30	42.20	36.60	3.48	3.76	4.33	3.11	3.22	3.18	–
Mean ± SD			33.5 ± 7.82	33 ± 8.48	162.25 ± 101.72	1.29 ± 0.30	1.23 ± 0.34	2.67 ± 0.81	41.98 ± 3.19	42.88 ± 1.09	34.08 ± 2.65	3.53 ± 0.11	3.32 ± 0.23	3.89 ± 0.47	3.14 ± 0.047	3.22 ± 0.03	3.23 ± 0.07	

Note: SM- *S. aureus* infected mastitis mothers; SMD- *S. aureus* infected daughters; HM- Healthy mothers; HMD- Healthy daughters; the four SMD animals were the descendants of the four SM animals, respectively; the four HMD animals were the descendants of the four HM animals, respectively; except HMD1 and HMD3 are half-siblings of one sire, the other animals are descendants of different sires; SCC: Somatic cell counts per milliliter of milk sample; SCS = log_2_(SCC/100,000) + 3; MY: milk yield, FP: milk fat percentage; PP: milk protein percentage; −indicates samples without *S. aureus* infection

For *S. aureus* identification, a total of 30 mL of fresh milk was collected and mixed from all of the four lactating quarters of each cow. Subsequently, based on the bacteriological culture and PCR results, four *S. aureus*-positive mother-daughter pairs were selected and separated into the *S. aureus* mother group (SM, *n* = 4) and the *S. aureus* daughter group (SMD, *n* = 4). Similarly, four *S. aureus*-negative mother-daughter pairs were separated into the healthy mother group (HM, *n* = 4) and the healthy daughter group (HMD, *n* = 4) (Fig. 1a).

### *S. aureus* isolation and identification

Bacteriological culture of milk samples was carried out according to National Mastitis Council standards [14]. A volume of 3 mL milk was mixed into trypticase soy broth containing 7.5% NaCl and cultured at 37 °C for 18–24 h. After that, a total of 10 mL culture was placed into Baird-Parker agar plates with tellurite and 5% egg yolk and cultured at 37 °C for 18–24 h. Two suspected colonies from each sample with surrounding clear zones were transferred to trypticase soy agar plated for DNA collection. Following this step, PCR amplification and sequencing of the *S. aureus* thermonuclease gene (*nuc*) [15] was performed (Fig. 1b and c). The PCR reaction was performed in 25 μL, containing 3 μL of genomic DNA (30–50 ng/μL), 1 μL of each primer (10 μmol), 12.5 μL of Taq™ Mix (1.25 units/25 μL reaction) and 7.5 μL of ddH_2_O. PCR was performed using the following thermocycler program: 94 °C for 10 min; 35 cycles of 94 °C for 30 s, 59 °C for 30 s and 72 °C for 30 s; 72 °C for 7 min.

### Blood collection, RNA extraction and RNA-sequencing

A 20-mL blood sample was obtained from the caudal vein from each animal for buffy coat (leukocytes) collection using 15 min centrifugation at 3,000 r/min.

TRIzol reagent (Invitrogen, Carlsbad, CA, USA) was used to isolate total RNA from leukocytes according to the manufacturer’s protocol. The RNA quality was checked on a 1% agarose gel and quantified using a Qubit RNA Assay Kit and a Qubit 2.0 Fluorometer (ThermoFisher Scientific, Waltham, MA USA). RNA integrity was assessed with the BioAnalyzer 2100 System (Agilent Technologies, Santa Clara, CA, USA). All 16 RNA samples had an RNA integrity number (RIN) larger than 7.0 (Supplementary Table S1). The 28S:18S rRNA ratios of all samples were larger than 1.7 (Supplementary Table S1). An equivalent amount (4 μg) of total PBL RNA purified from each animal was used to construct RNA-Seq libraries with the NEBNext® Ultra™ RNA Library Prep Kit for Illumina® (NEB, Ipswich, MA, USA). Finally, the libraries were sequenced using 150 bp paired-end reads with the Illumina HiSeq X Ten System (CapitalBio Technology, Beijing, China).

### Quality control for raw data

Trimmomatic software version 0.38 [16] was used to filter out the adapter sequence and low-quality bases/reads with the default parameters (http://www.usadellab.org/cms/?page=trimmomatic). Further quality assessment of the sequence reads was then undertaken using FastQC version 0.11.8 [17]. After these QC procedures were completed, the sequence read data were used for the downstream analyses in the computational workflow.

### Reads alignment and abundance estimation

The *Bos taurus* ARS-UCD1.2 reference assembly (FASTA format) and annotated gene model (GTF format) were downloaded from the Ensembl database (ftp.ensemble.org/pub/release-96/gtf/bos_taurus). The QC-assessed sequence read data for each sample were aligned to the reference genome using STAR with the basic options [18]. Transcript abundance was quantified using featureCounts [19] in the R subread package [20] under the default setting, and read counts were calculated to estimate the transcript expression levels. Genes with read counts more than ten in at least two samples were defined as expressed genes. The rlog-normalized read count was calculated with DESeq2 [21], which was then used to performed differential expression analysis.

The transcriptional responses to *S. aureus* infection were investigated by comparing differentially expressed genes between the infected and non-infected healthy control groups. We firstly analyzed gene expression levels in the S group (*n* = 8, 4 animals each in the SM and SMD groups) and H group (*n* = 8, 4 animals each in the HM and HMD groups), and compared the differentially expressed genes (DEGs) between the two groups. Detection of DEGs was also performed for the comparisons of the mother groups (SM vs. HM) and the daughter groups (SMD vs. HMD).

### Functional enrichment and annotation

Gene set enrichment analysis was performed for all the expressed genes detected between the S and H groups, using the GSEA software package (v4.0.3) with the Human NCBI Gene ID Molecular Signature Database (MSigDB) version 7.0 and Hallmark database version 7.0 [22–24]. Gene sets were considered significant when *P* <  0.05 and *FDR* <  0.25 [25, 26].

The DEGs (*P* <  0.05 and |FC| > 2) in the comparisons of S vs. H, SM vs. HM and SMD vs. HMD were used to perform the following functional enrichment analysis. Kyoto Encyclopedia of Genes and Genomes (KEGG) pathway and Gene Ontology (GO) enrichment analyses of gene clusters were performed using clusterProfiler [27], with results exhibiting *P* <  0.05 considered significant. An interaction network analysis was also conducted using Ingenuity Pathway Analysis (IPA; Qiagen, Redwood City, CA, USA) [28].

### Validation of RNA-Seq results through reverse transcription quantitative real-time PCR (RT-qPCR)

To quantitatively assess the reliability of our sequencing data, the expression levels of seven (SM vs. HM comparison) and ten (SMD vs. HMD comparison) randomly selected genes were estimated with RT-qPCR in the same samples used for RNA-Seq (Supplementary Table S1). The RNA samples were reverse transcribed into cDNA using the PrimeScript™ RT reagent kit according to the manufacturer’s instructions (Takara Bio, Dalian, China). Real-time detection of specific PCR products was performed with the Sybr Green I Master Mix Kit (Roche Diagnostics, Mannheim, Germany) on the LightCycler 480 II (Roche Diagnostics Ltd., Basel, Swetzerland), according to the manufacturer’s protocol. The oligonucleotide primers used for the RT-qPCR analysis are provided in Supplementary Table S2. The thermocycler program used was as follows: one cycle of preincubation at 95 °C for 10 min, 45 cycles of amplification (95 °C for 10 s, 60 °C for 10 s, and 72 °C for 10 s). Duplicate RT-qPCR assays were performed on each cDNA sample and relative expression values were calculated using the 2^–△△Ct^ method with the bovine glyceraldehyde-3-phosphate dehydrogenase gene (*GAPDH*) as the internal reference [29, 30]. The log_2_|FC| values were calculated from RT-qPCR data to directly compare to the RNA-Seq results.

### Statistical analysis

A linear regression analysis was conducted using the Prism software package (version 8; GraphPad Software, San Diego, CA, USA) to evaluate gene expression levels among experimental groups. For RT-qPCR data, the significant differences between groups were examined with the Student’s *t*-test.

## Results

### Transcriptomic data and detection of expressed genes in peripheral blood leukocyte samples

**Fig. 2:**
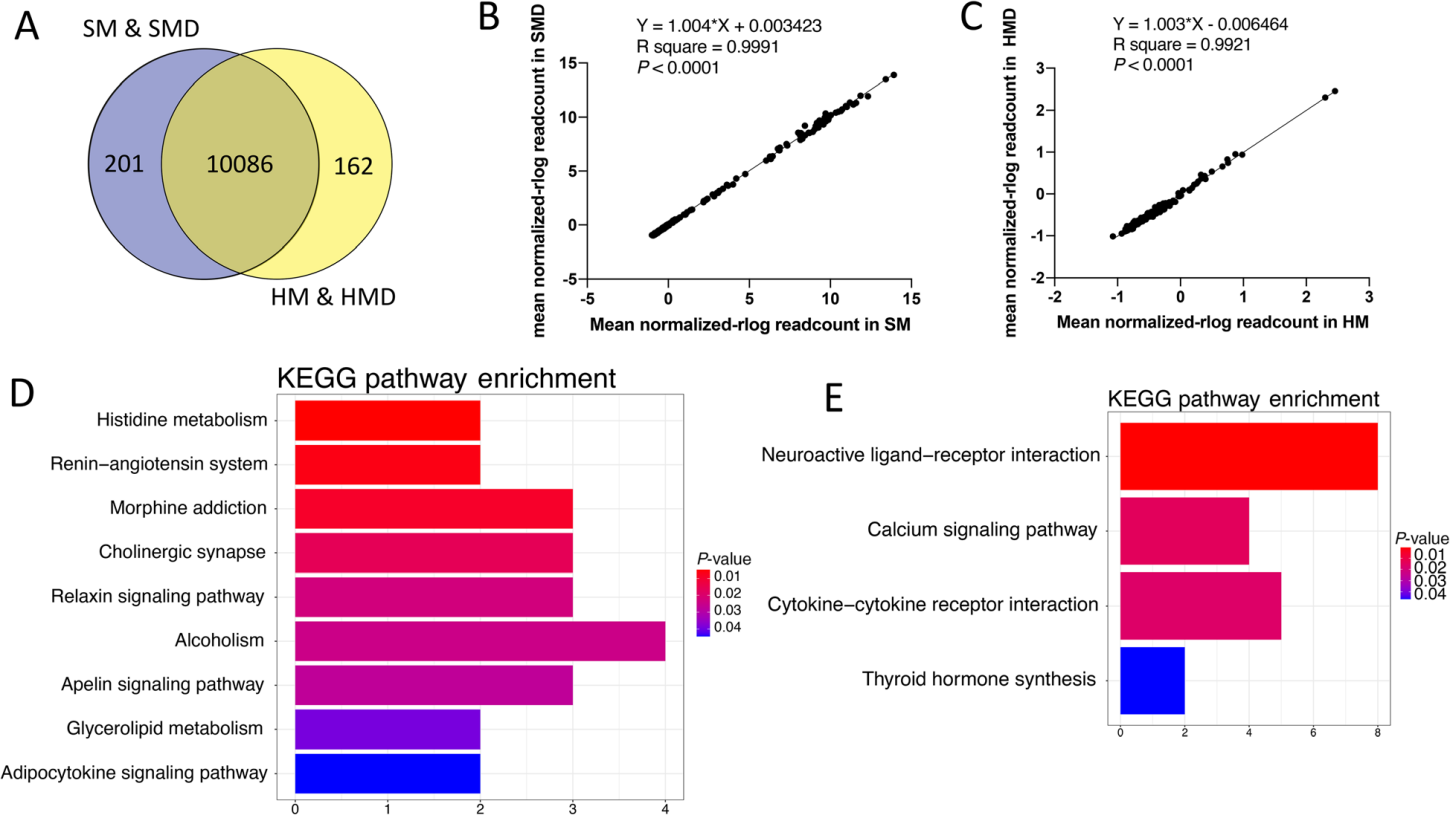
Comparison of gene expression profiles between mothers and daughters. **a** Venn diagram showing genes only expressed in *S. aureus*-positive mother-daughter pairs (purple circle), genes only expressed in *S. aureus*-negative mother-daughter pairs (yellow circle), and genes in intersection are common to both groups. **b** Linear regression analysis of expression levels for 201 genes exclusively expressed in the SM and SMD groups. **c** Linear regression analysis of expression levels for 162 genes exclusively expressed in the HM and HMD groups. **d** Significantly enriched (*P* < 0.05) KEGG pathways of genes exclusively expressed in the *S. aureus*-positive groups (SM and SMD). **e** Significantly enriched (*P* < 0.05) KEGG pathways of genes exclusively expressed in the *S. aureus*-negative groups (HM and HMD)

In the present study, an average of 22,435,225 (ranging from 18,801,274 to 25,620,906) paired-end reads were generated as raw data from the 16 RNA-Seq libraries. After filtering, an average of 20,955,429 reads (ranging from 16,864,277 to 23,829,973) were retained for each library. The average uniquely mapping rate was 94.11% (Supplementary Table S3). There were 10,287 detectable genes expressed in both the SM and SMD groups, and 10,248 gene expressed in the HM and HMD groups (Fig. 2a). A total of 10,086 detectable expressed genes were shared across the four groups. A total of 201 genes were identified that were exclusively expressed in the two consecutive generations of animals naturally infected with *S. aureus* (SM and SMD), which may be associated with susceptibility to *S. aureus*-induced mastitis. Conversely, 162 genes were exclusively expressed in the two consecutive generations of healthy control animals (HM and HMD), which may be associated with resilience to *S. aureus*-induced mastitis.

Furthermore, the correlation between the gene expression profiles (log_2_|FC| of 4000 randomly selected genes) in the two generations of SM vs. HM and SMD vs. HMD was significant (*R*^2^ = 0.3410, *P* < 0.0001, Supplementary Fig. S1). This result indicates that gene expression changes due to *S. aureus* infection challenge were moderately conserved between the two generations. The linear regression analyses were also performed using the normalized read counts of the 201 exclusively expressed genes (*R*^2^ = 0.9991, *P* < 0.0001, Fig. 2b) in the two generations of animals naturally infected with *S. aureus* (SM and SMD) and the 162 exclusively expressed genes (*R*^2^ = 0.9921, *P* < 0.0001, Fig. 2c) in the two generations of healthy controls (HM and HMD).

### Transcriptome changes in peripheral blood leukocytes from *S. aureus*-positive cattle compared to *S. aureus*-negative control cattle

**Fig. 3:**
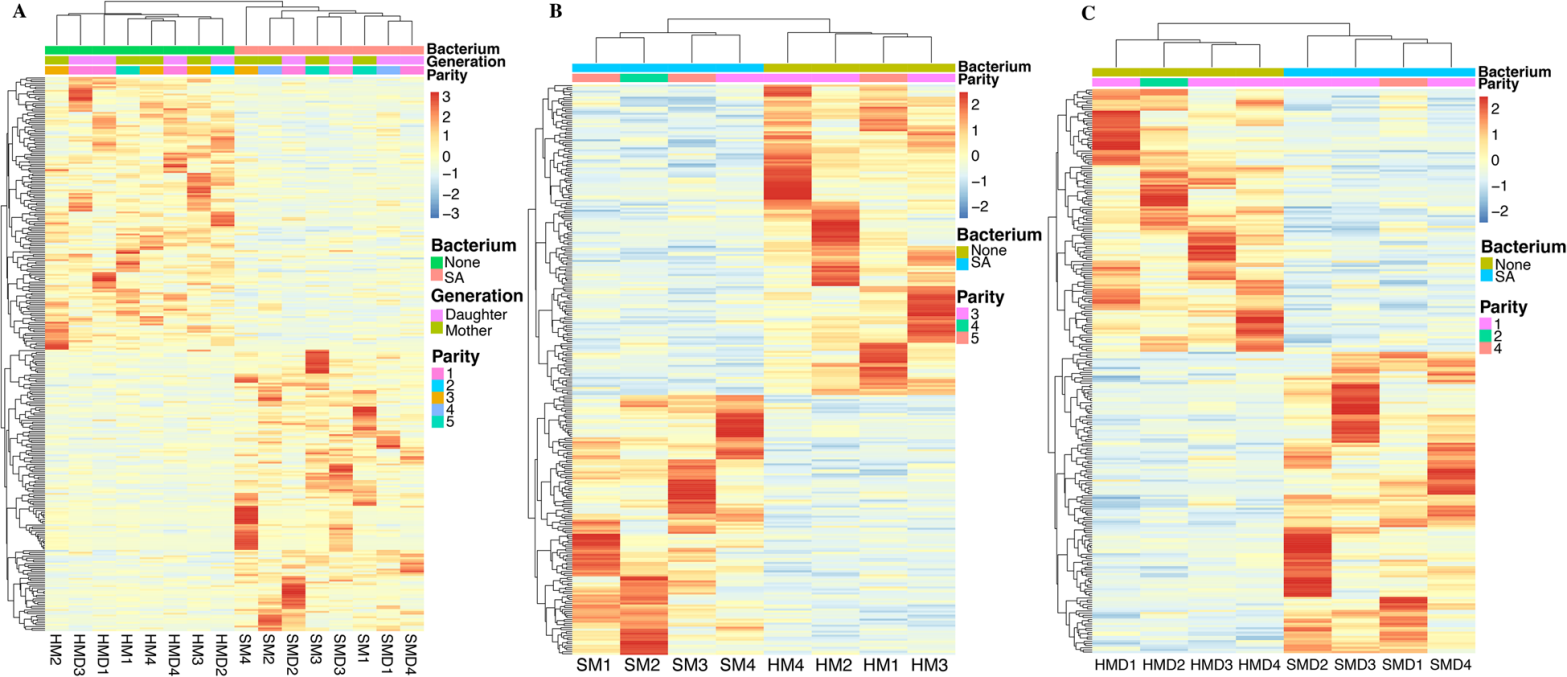
Transcriptomic changes of PBL in the three comparisons. Cluster analyses and heat maps of DEGs in (**a**) S vs. H, (**b**) SM vs. HM and (**c**) SMD vs. HMD. The different columns represent different samples and the different rows denote different DEGs. Red and blue show increased and decreased expression, respectively

**Fig. 4:**
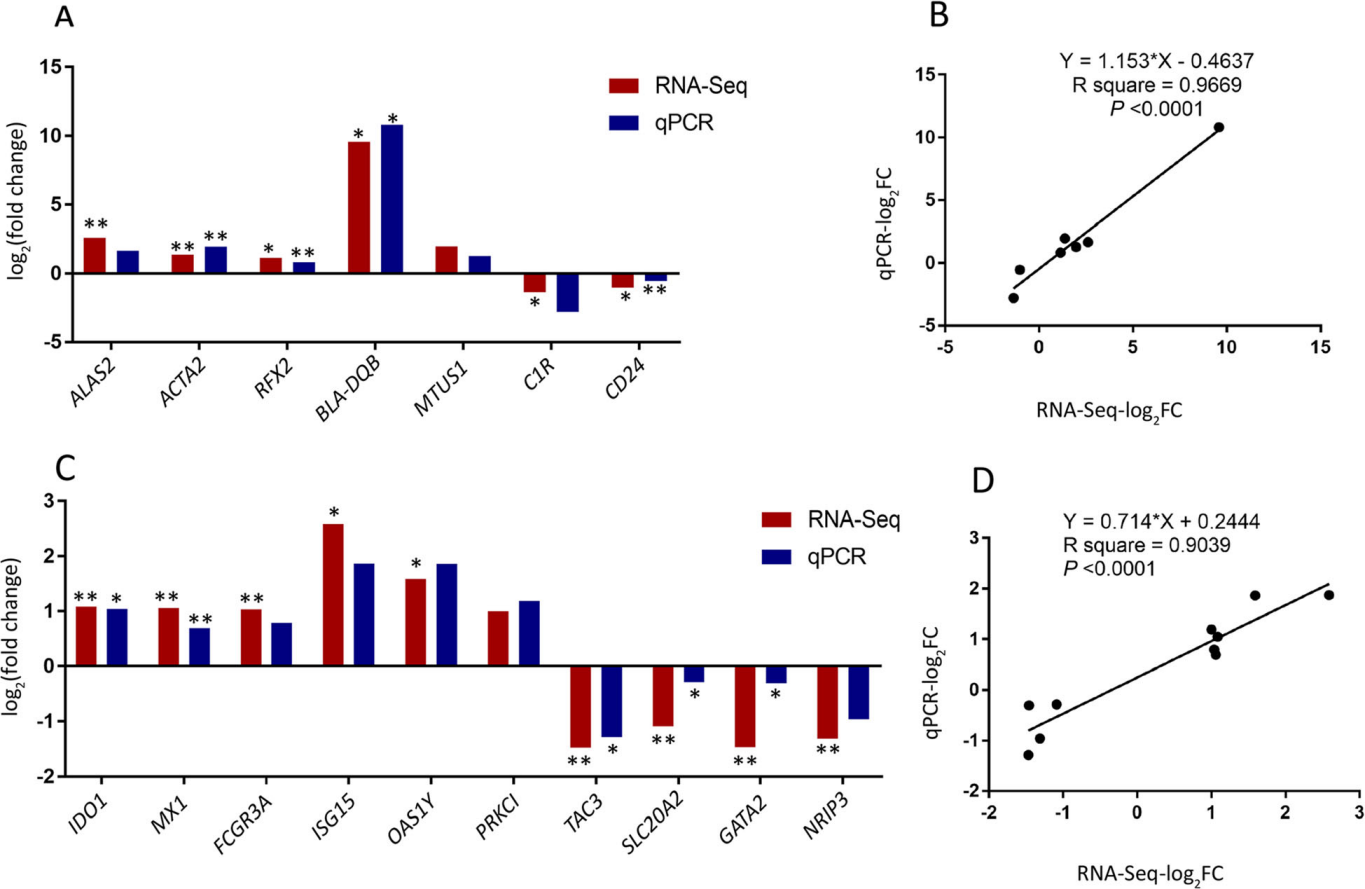
RT-qPCR validation of RNA-Seq results. **a** and **c** RT-qPCR confirmation results for the randomly selected DEGs from the SM vs. HM comparison and the SMD vs. HMD comparison, respectively. **b** and **d** Regression analysis of the log_2_|FC| values between the RNA-Seq and RT-qPCR validation of the SM vs. HM and the SMD vs. HMD comparisons, respectively. The *GAPDH* gene was used as an internal reference control gene. * means *P* < 0.05. ** means *P* < 0.01

It is well established that *S. aureus*, as an intracellular pathogen of mammals, has evolved a wide range of mechanisms for immunosuppression and immunoreaction [31, 32]. To investigate host-pathogen interaction in bovine PBL infected with *S. aureus*, the transcriptomes of infected cattle and non-infected healthy control animals were compared. Although no significant DEGs were detected using an FDR *P*_adj._ threshold of 0.05, a total of 17, 13 and 10 DEGs were observed for the three comparisons (Table 2, 3 and 4), respectively, using the criteria of FDR *P*_adj._ < 0.1 and |FC| > 1.2 [33–35]. Furthermore, a total of 301, 283 and 260 DEGs (*P* < 0.05, log_2_|FC| > 2) (Supplementary Table S5, 6 and 7 and Fig. S2) were used for functional enrichment analysis. The expression heat maps of the 301, 283 and 260 genes of the three comparisons revealed different transcriptional profiles between *S. aureus*-positive and *S. aureus*-negative samples (Fig. 3). To validate the RNA-Seq results, seven and ten genes were selected for RT-qPCR validation in the SM vs. HM and SMD vs. HMD comparisons (Fig. 4), respectively. Results from this analysis showed that the gene expression patterns obtained using RNA-Seq were consistent with the results generated from RT-qPCR (Fig. 4a and c). The correlation coefficients between the RNA-Seq and RT-qPCR results for the genes in the SM vs. HM and SMD vs. HMD comparisons were 0.97 and 0.90 (*P* < 0.0001), respectively (Fig. 4b and d).

**Table 2:** Differentially expressed genes in the S vs. H comparison (FDR *P*_adj._ < 0.1 with |FC| > 1.2)

Ensembl ID	Gene symbol	Genome position	Log_2_ fold change	*P*-value	*P*_adj._
ENSBTAG00000006859	*CEACAM6*	18:51400717–51406445	−1.5900	4.67E-10	8.06E-06
ENSBTAG00000037452	*LOC790312*	10:26644523–26648616	−2.8690	1.16E-07	0.0010
ENSBTAG00000043548	*–*	MT:364–430	3.3845	3.37E-06	0.0194
ENSBTAG00000005078	*UCHL1*	6:60147025–60159287	1.1728	5.91E-06	0.0255
ENSBTAG00000017786	*FCRL6*	3:9870119–9891157	2.1591	8.41E-06	0.0290
ENSBTAG00000048420	*–*	18:62937680–62942083	− 1.6206	1.24E-05	0.0340
ENSBTAG00000017038	*HPS4*	17:66113220–66140520	−0.5173	1.38E-05	0.0340
ENSBTAG00000018596	*PTPN21*	10:100170718–100247414	−1.488	1.59E-05	0.0344
ENSBTAG00000047621	*–*	16:5146970–5149550	0.9748	1.84E-05	0.0352
ENSBTAG00000045507	*ZNF469*	18:13698486–13709861	−1.5298	4.01E-05	0.0691
ENSBTAG00000005725	*IDO2*	27:35048477–35114325	1.6464	5.64E-05	0.0886
ENSBTAG00000013281	*SPATA21*	2:135698556–135736414	−0.6353	6.17E-05	0.0887
ENSBTAG00000019455	*MYO5B*	24:49490278–49824858	−1.1553	7.22E-05	0.0958
ENSBTAG00000011554	*SURF6*	11:104240172–104246931	−0.4955	8.39E-05	0.0997
ENSBTAG00000053500	*–*	5:74430343–74435609	−1.0656	9.18E-05	0.0997
ENSBTAG00000049399	*–*	4:82745643–82745786	1.2984	9.35E-05	0.0997
ENSBTAG00000035945	*–*	4:50056455–50082481	1.0603	9.82E-05	0.0997

**Table 3:** Differentially expressed genes in the SM vs. HM comparison (FDR *P*_adj._ < 0.1 and |FC| > 1.2)

Ensembl ID	Gene symbol	Genome position	Log_2_ fold change	*P*-value	*P*_adj._
ENSBTAG00000006859	*CEACAM6*	18:51400717–51406445	−2.1830	3.54E-10	5.26E-06
ENSBTAG00000015405	*DCHS1*	15:46407409–46444149	1.1298	8.23E-08	0.0006
ENSBTAG00000039046	*CD24*	2:117785379–117787180	−1.3277	6.26E-06	0.0311
ENSBTAG00000018077	*LYPD3*	18:51674245–51678673	1.4669	1.53E-05	0.0568
ENSBTAG00000035945	*–*	4:50056455–50082481	1.5454	2.57E-05	0.0723
ENSBTAG00000054978	*–*	4:99079895–99088132	1.1172	3.14E-05	0.0723
ENSBTAG00000015061	*–*	18:61209482–61224598	2.2668	3.40E-05	0.0723
ENSBTAG00000000507	*NR4A1*	5:27820352–27839685	−0.8279	4.84E-05	0.0843
ENSBTAG00000053508	*–*	21:312642–314960	−2.6172	5.30E-05	0.0843
ENSBTAG00000017786	*FCRL6*	3:9870119–9891157	2.9479	5.67E-05	0.0843
ENSBTAG00000053424	*–*	7:5751646–5755931	−2.1134	6.96E-05	0.0942
ENSBTAG00000047302	*–*	13:67292055–67307842	2.3906	7.65E-05	0.0949
ENSBTAG00000046383	*–*	18:62453964–62454894	−2.0405	8.60E-05	0.0984

**Table 4:** Differentially expressed genes in the SMD vs. HMD comparison (FDR *P*_adj._ < 0.1 and |FC| > 1.2)

Ensembl ID	Gene symbol	Genome position	Log_2_ fold change	*P*-value	*P*_adj._
ENSBTAG00000037452	*LOC790312*	10:26644523–26648616	−3.2530	5.89E-07	0.0066
ENSBTAG00000043250	*7SK*	23:25224255–25224585	4.4505	7.57E-07	0.0066
ENSBTAG00000053635	*–*	21:394145–395961	−2.0210	2.72E-06	0.0158
ENSBTAG00000019741	*C3AR1*	5:101566575–101575901	2.4457	5.54E-06	0.0242
ENSBTAG00000006582	*SSTR1*	21:48348493–48351072	−3.1215	9.41E-06	0.0300
ENSBTAG00000039440	*–*	19:20556890–20557410	5.8616	1.03E-05	0.0300
ENSBTAG00000019455	*MYO5B*	24:49490278–49824858	−1.4318	1.92E-05	0.0480
ENSBTAG00000048135	*–*	20:71927027–71928602	−1.5261	2.76E-05	0.0603
ENSBTAG00000050180	*Metazoa_SRP*	10:42834493–42834792	5.9138	3.30E-05	0.0641
ENSBTAG00000008103	*ALDH1A1*	8:49053228–49106706	3.0839	3.70E-05	0.0646

### Functional enrichment and annotation

The GSEA analysis performed between S and H groups revealed that a total of 38 gene sets were upregulated in S group compared to the controls, of which six gene sets were significant at *FDR* < 0.25 and *P* < 0.05. Significantly enriched gene sets (*FDR* < 0.25 and *P* < 0.05) in the S group are shown in Supplementary Fig. S3 and the enrichment information is summarized in Supplementary Table S4.

KEGG pathway analysis of 201 genes exclusively expressed in *S. aureus*-positive animals revealed nine significantly enriched KEGG pathways (*P* < 0.05) (Fig. 2d). Whereas genes exclusively expressed in *S. aureus*-negative animals revealed four significantly enriched KEGG pathways (*P* < 0.05) (Fig. 2e). Five genes (*EPOR*, *IL9*, *IFNL3*, *CCL26*, and *IL26*) exclusively expressed in *S. aureus*-negative animals were enriched in the cytokine-cytokine receptor interaction KEGG pathway.

**Fig. 5:**
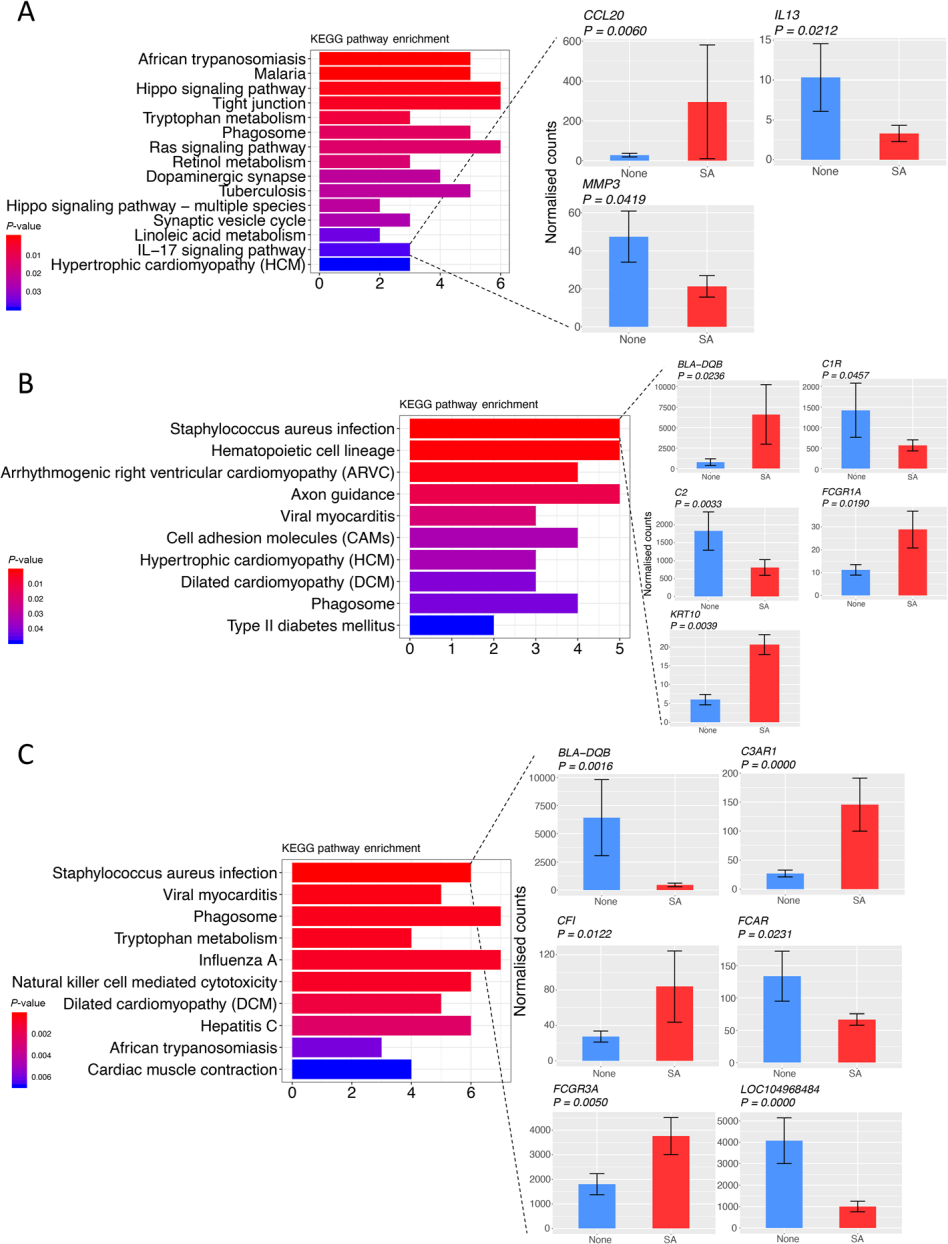
KEGG pathway enrichment of DEGs. **a** KEGG pathways of DEGs in the comparison of S vs. H and expression differences of the DEGs involved in the enrichment of the IL-17 signaling pathway. **b** KEGG pathways of DEGs in the comparison of SM vs. HM and expression differences of the DEGs involved in the enrichment of the *S. aureus* infection pathway. **c** KEGG pathways of DEGs in the comparison of SMD vs. HMD and expression differences of the DEGs involved in the enrichment of the *S. aureus* infection pathway

KEGG pathway analysis of the 301 DEGs in the S vs. H comparison revealed 15 significant enriched pathways (*P* < 0.05) (Fig. 5a). Most of these pathways were associated with the immune response or inflammation. For example, the IL-17 signaling pathway was enriched by three DEGs (*CCL20*, *IL13,* and *MMP3*). Furthermore, ten KEGG pathways were significantly enriched (*P* < 0.05) by DEGs in the SM vs. HM comparison and, among these, *S. aureus* infection was the most significantly enriched pathway (Fig. 5b). Moreover, DEGs in the comparison of SMD vs. HMD were significantly (*P* < 0.05) enriched in ten pathways, including *S. aureus* infection, phagosome. Importantly, six DEGs were significantly involved in the *S. aureus* infection pathway (Fig. 5c). The top ten biological processes identified using GO enrichment analysis are shown in Supplementary Fig. S4.

**Fig. 6:**
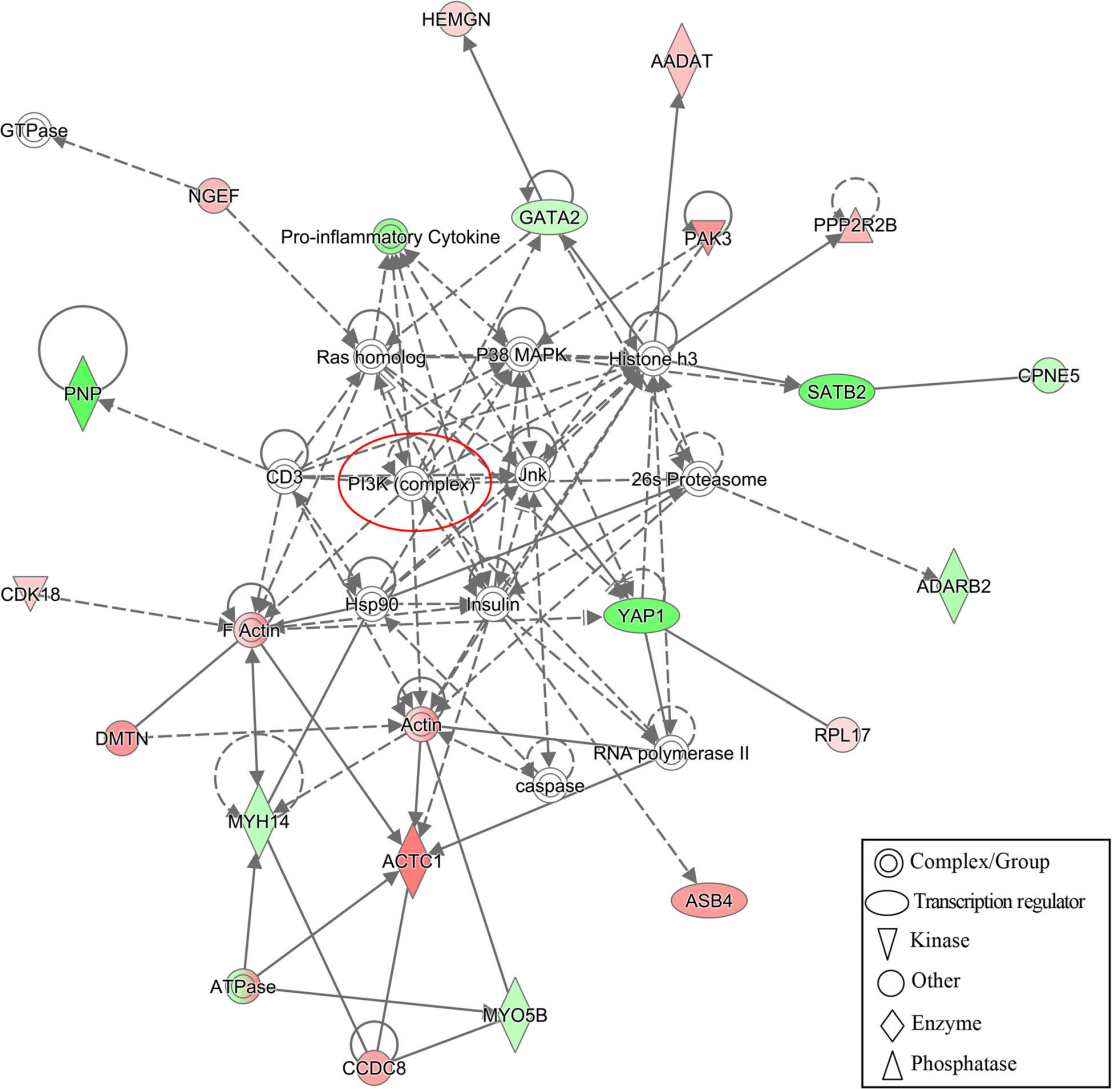
IPA interaction network generated from the DEG list in the S vs. H comparison. Red denotes gene nodes that were increased in expression, and green denotes nodes that were decreased in expression in S samples compared to H samples. The red circle denotes a commonly shared node in the three comparisons

**Fig. 7:**
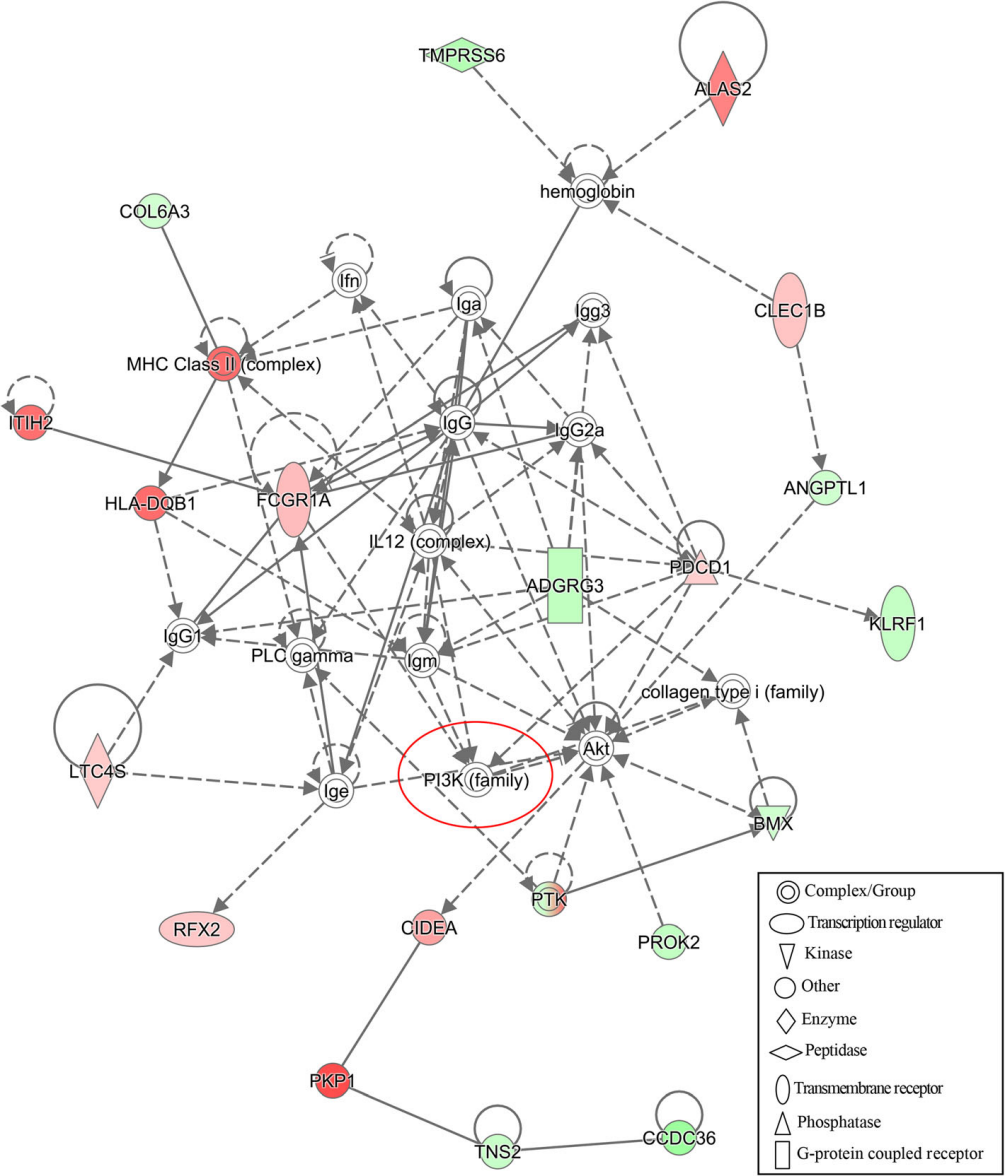
IPA interaction network generated from the DEG list in the SM vs. HM comparison. Red denotes gene nodes that were increased in expression, and green denotes nodes that were decreased in expression in SM samples compared to HM samples. The red circle denotes a commonly shared node in the three comparisons

**Fig. 8:**
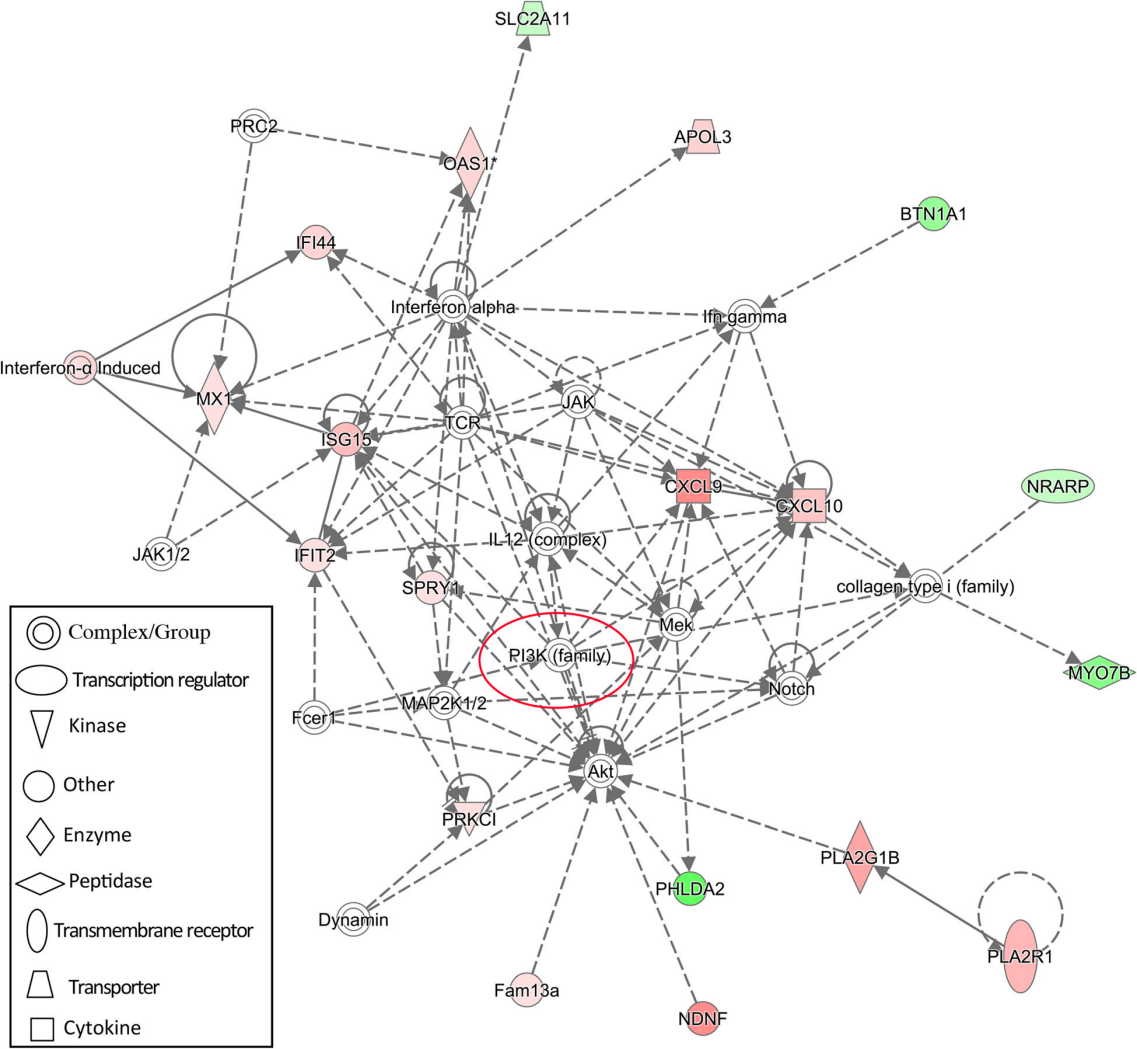
IPA interaction network generated from the DEG list in the SMD vs. HMD comparison. Red denotes gene nodes that were increased in expression, and green denotes nodes that were decreased in expression in SMD samples than HMD samples. The red circle denotes a commonly shared node in the three comparisons

To construct interaction networks, the DEGs in each comparison were further analyzed using the IPA software tool. Interestingly, the PI3K family is shared across all networks generated for the three comparisons and interacted with 13, 6 and 11 molecules in comparisons of S vs. H, SM vs. HM and SMD vs. HMD, respectively (Figs. 6, 7 and 8). Moreover, the IL12 complex and kinase AKT both play key roles in the SM vs. HM and SMD vs. HMD comparison (Figs. 7 and 8). Collectively, these functional analysis results suggested that the immune responses were activated in PBL from *S. aureus*-infected dairy cattle.

**Fig. 9:**
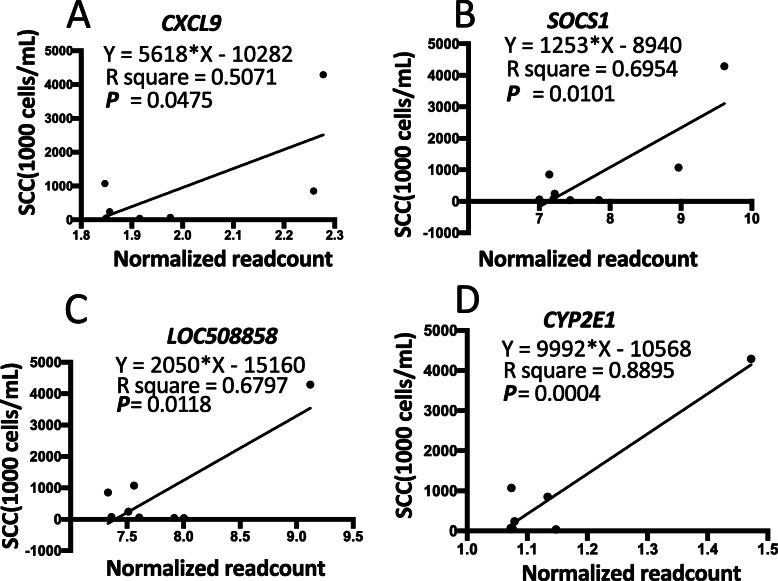
Linear regression analysis between normalized read counts and SCC. The *X*-axis indicates normalized read counts of DEGs, and the *Y*-axis indicates SCC. **a**-**d**) represent *CXCL9, SOCS1, LOC508858,* and *CYP2E1*, respectively

Finally, the linear regression results between normalized read counts and SCC showed that significant correlations were observed between the expression levels of DEGs (*CXCL9*, *SOCS1*, *LOC508858*, and *CYP2E1*) and SCC (Fig. 9), suggesting these genes might be served as potential molecular biomarkers of mastitis caused by infection with *S. aureus*.

## Discussion

In recent years, health traits, including mastitis resistance, are increasingly being incorporated into the breeding goals of the modern dairy industry [36–38]. Mastitis is a low heritability trait [39, 40] and it is therefore difficult to achieve significant improvements using conventional breeding strategies. Therefore, it is important to investigate the complex host-pathogen interaction underlying mastitis disease caused by *S. aureus* infection from the perspective of the peripheral blood transcriptome. In previous genome-wide association studies (GWAS), a number of candidate genes (*TRAPPC9* [41], *mTORC1* [42], *JAK2* and *STAT5A* [43]) were observed to be associated with mastitis-related traits such as SCC. However, transcriptome profiles of *S. aureus* infection in both cows and their offspring have not been studied in detail. In the current study, for the first time, transcriptome profiles of PBL in response to *S. aureus* infection were investigated in two generations of dairy cattle.

Previous studies have identified many genes as potential expression biomarkers for bovine mastitis caused by infection with *S. aureus* mastitis [6, 44]. However, the concordance among these results is not high, possibly as a consequence of environmental factors and varying genetic backgrounds. To address this issue, eight mother-daughter pairs (four *S. aureus*-infected pairs and four uninfected mother-daughter pairs) from the same farm were used as experimental subjects for the current study. Three well-defined animal selection criteria were used for comparative analyses of the transcriptomes of PBL sampled from two generations of dairy cattle infected with *S. aureus*-induced mastitis: 1) availability of mother-daughter pairs, 2) three consecutive months of SCC score recordings, and 3) unambiguous *S. aureus* detection and identification.

For dairy cattle, to produce 1 kg of milk, 400 to 500 kg of blood must pass through the udder [45]. Consequently, PBL represents a valuable sample resource to evaluate the condition of udder health. In our previous study [44], we infected udder quarters with *S. aureus* (1 × 10^6^ CFU/mL), then conducted mammary biopsies, and collected udder tissues for RNA extraction and transcriptome analysis using RNA-Seq. Mammary biopsies cause trauma and stress; therefore, the use of an accessible and informative tissue such as peripheral blood from naturally infected animals in a production population substantially mitigates animal welfare issues. Thus, PBL samples from two generations of *S. aureus*-infected cows were used to perform transcriptome sequencing.

Our study is the first to describe transcriptomic profiles of PBL in *S. aureus*-infected and uninfected dairy cattle across two generations. More than 160 genes were observed solely in the healthy mother-daughter pairs compared to the *S. aureus*-infected mother-daughter pairs, an observation that may be relevant to identification of genes underpinning resilience to *S. aureus* mastitis (Fig. 2a). These genes were observed to be significantly enriched in four biological pathways with the cytokine-cytokine receptor interaction pathway directly related to the immune response. Five genes (*EPOR*, *IL9*, *IFNL3*, *CCL26*, and *IL26*) that were involved in this pathway might serve as potential molecular markers for breeding programs that enhance resistance to *S. aureus* infection and mastitis (Fig. 2e). Thus, these immune-related genes warrant further study in a larger group of animals across two generations.

*EPOR* encodes the erythropoietin receptor, which is a member of the cytokine receptor family. It can activate JAK2 tyrosine kinase that, in turn, activates a range of intracellular pathways. A previous study observed that the *IL9* gene emerged as a central node in the network associated with *Streptococcus agalactiae-*induced subclinical mastitis [46]. In this regard, we have also identified *IL9* as a key gene associated with *S. aureus*-induced subclinical mastitis. Interestingly, sequence variation at *IFNL3* has been shown to be associated with hepatitis B virus (HBV) infection in a Chinese human population [47]. *CCL26* encodes a secreted protein involved in immunoregulatory and inflammatory processes, which Anna et al. observed to be associated with IL4-mediated signaling pathways in bovine macrophages challenged *in vitro* with *S. agalactiae* [48]. The protein encoded by *IL26* is a member of the IL10 family of cytokines [49], which has been shown to exhibit anti-bacterial activity against a wide range of bacteria including *S. aureus* [50], suggesting a broader role in host defenses against bacteria [51].

As expected, a small number of DEGs (Fig. 3) were detected using FDR *P*_adj._ < 0.1, a less stringent statistical threshold. Chinese dairy cattle represent a relatively outbred animal population with significant variation in genetic background [52]. *S. aureus* can readily adapt to its host through evasion of almost every facet of the immune system [32]; therefore, *S. aureus*-infected cows may exhibit less severe and milder immune responses, which may account for the relatively small number of DEGs. In this regard, Fang et al. also reported modest differential gene expression in bovine mammary gland induced by a low dose of *S. aureus* to simulate naturally infected mastitis [44]. It would therefore be instructive to perform a substantially larger *in vitro* challenge experiment using varying *S. aureus* challenge doses.

Indoleamine 2,3-dioxygenase 2 encoded by *IDO2* is an immunomodulatory molecule with potential effects on various diseases including cancer and autoimmune conditions. A previous study demonstrated that *IDO2* functions as a modifier in B cells to control pathogenic inflammation and autoimmunity [53]. *NR4A1*, which encodes the nuclear receptor subfamily 4 group A member 1 protein, was differentially expressed in the comparison of SM vs. HM for the current study. *NR4A1* has been shown to play a key role as a regulator of the immune response to apoptotic cells [54].

In the present study, immune-related pathways were enriched by different but complementary functional enrichment and annotation methods (KEGG, IPA and GSEA). Three DEGs (*CCL20*, *IL13,* and *MMP3*) were found to be involved in the IL-17 signaling pathway in the S vs. H comparison (Fig. 5a). The interleukin 17 (IL-17) family are proinflammatory cytokines that play crucial roles in both acute and chronic inflammatory responses [55]. Moreover, there are five (*BLA-DQB*, *C1R*, *C2*, *FCGR1A*, and *KRT10*) (Fig. 5b) and six genes (*BLA-DQB*, *C3AR1*, *CFI*, *FCAR*, *FCGR3A*, and *LOC10498484*) (Fig. 5c) enriched in the *S. aureus* infection pathway in the SM and SMD groups, respectively. The *BLA-DQB* gene encodes the bovine major histocompatibility complex, class I, DQ beta protein, which has been hypothesized to play a key role in the synchronization of the immune response to mastitis-causing bacteria in bovine udder secretory tissues [56]. Interestingly, *BLA-DQB* exhibited contrary directions of expression in SM and SMD compared with the healthy controls, which might be due to the differences in the duration of *S. aureus* infection [57], differences in age [58], parity [59] and lactation period [35] of the cattle.

The IPA analysis revealed an interaction network involving the phosphoinositide 3-kinase (PI3K) family in all three comparisons (Fig. 6, 7 and 8). The PI3K family of enzymes are involved in myriad cellular functions, including cell growth, proliferation, differentiation, motility, survival, and intracellular trafficking. In particular, PI3K proteins have important functions in immunobiology [60]. *IL12* (Fig. 7 and 8) encodes an important cytokine that can regulate both innate and adaptive immune responses during infection [61]. Kinase AKT (Fig. 7 and 8) is involved in the regulation of the development and function of innate immune cells, including neutrophils, macrophages, and dendritic cells [62].

The cytokine-cytokine receptor signaling pathway genes exclusively expressed in uninfected mother-daughter pairs (*EPOR*, *IL9*, *IFNL3*, *CCL26*, and *IL26*) and the DEGs involved in immune-related pathways identified in the current study (including *CCL20*, *IL13, MMP3,* and *BLA-DQB*), may contain sequence polymorphisms that could serve as genetic markers for susceptibility to mastitis caused by *S. aureus* infection. Consequently, identification of SNPs within these genes and systematic evaluation through large-scale association studies using suitable animal cohorts can provide valuable information for future genome-enabled breeding programs to enhance mastitis resilience in dairy cattle.

## Conclusions

In summary, the transcriptome profiles of PBL sampled from two consecutive generations of cows with naturally infected *S. aureus* mastitis and non-infected healthy control animals were investigated. Many of the genes highlighted in this study as being important in the bovine host response to infection with *S. aureus* may represent candidate PBL expression biomarkers for mastitis and may also contain sequence variation that can be leveraged for genomic selection of cattle less susceptible to mastitis disease.

## Supplementary information

**Additional file 1: Supplementary Figure S1.** Linear regression analysis of the log_2_|FC| of 4000 randomly selected gene expression values for the SM vs. HM and SMD vs. HMD comparisons.

**Additional file 2: Supplementary Figur S2.** Volcano plots of DEGs for (A) the S vs. H comparison, (B) the SM vs. HM comparison, and (C) the SMD vs. HMD comparison.

**Additional file 3: Supplementary Figure S3.** Gene set enrichment analysis (GSEA) plots depicting the enrichment of functional gene sets up-regulated in the S group compared to the control group (*FDR* < 0.25 and *P* < 0.05). (A-F): GSEA plot depicting the enrichment of functional gene sets up-regulated in oxidative phosphorylation, heme metabolism, fatty acid metabolism, Kras signaling up-regulation, inflammatory response and interferon-gamma response in the S vs. H comparison (*FDR* < 0.25 and *P* < 0.05).

**Additional file 4: Supplementary Figure S4.** GO Biological Process enrichment of DEGs. (A) Top ten Biological Process GO categories of DEGs in the S vs. H comparison. (B) Top ten Biological Process GO categories of DEGs in the SM vs. HM comparison. (C) Top ten Biological Process GO categories of DEGs in the SMD vs. HMD comparison.

**Additional file 5: Table S1.** Quality report of 16 RNA samples for RNA sequencing.

**Additional file 6: Table S2.** Primer pairs of DEGs used for qRT-PCR validation.

**Additional file 7: Table S3.** Summary of sequence reads aligenment

**Additional file 8: Table S4.** Six significantly enriched upregulated gene sets.

**Additional file 9: Table S5.** Differentially expressed genes detected in S vs. H

**Additional file 10: Table S6.** Differentially expressed genes detected in SM vs. HM.

**Additional file 11: Table S7.** Differentially expressed genes detected in SMD vs. HMD.

## Data Availability

All genomic annotation data defining gene regions are available for download (ftp://ftp.ensemble.org/pub/release-96/gtf/bos_taurus). RNA-Seq data from China Agricultural University is available upon the agreement of China Agricultural University and should be requested directly from the authors.

## Abbreviations

*S. aureus*: *Staphylococcus aureus*
S: *S. aureus*-infected cows
H: Healthy non-infected cows
SM: *S. aureus*-infected mastitis mothers
HM: Healthy non-infected mothers
SMD: *S. aureus*-infected daughters
HMD: Healthy non-infected daughters
EPOR: Erythropoietin receptor
IL9: Interleukin 9
IFNL3: Interferon lambda 3
CCL26: C-C motif chemokine ligand 26
IL26: Interleukin 26
DEGs: Differentially expressed genes
FDR: False discovery rate
CCL20: Chemokine (C-C motif) ligand 20
IL13: Interleukin 13
MMP3: Matrix metalloproteinase-3
IL-17: Interleukin 17
BLA-DQB: *Bos taurus* major histocompatibility complex, class II, QB beta
C1R: Complement C1r subcomponent
C2: Complement C2
FCGR1A: Fc fragment of IgG receptor Ia
KRT10: Keratin 10
C3AR1: Complement component 3a receptor 1
CFI: Complement factor I
FCAR: Fc fragment of IgA receptor
FCGR3A: Fc fragment of IgG, low affinity IIIa, receptor
*E. coli*: *Escherichia coli*
RNA-Seq: RNA sequencing
HTS: High-throughput sequencing
LPS: Lipopolysaccharide
PBL: Peripheral blood leukocyte
SCC: Somatic cell counts
DHI: Dairy herd improvement
Nuc: Thermonuclease
PCR: Polymerase chain reaction
RIN: RNA integrity number
GSEA: Gene set enrichment analysis
MSigDB: Molecular signature database
KEGG: Kyoto Encyclopedia of Genes and Genomes
GO: Gene ontology
IPA: Ingenuity Pathway Analysis
RT-qPCR: Reverse transcription quantitative real-time PCR
GAPDH: Glyceraldehyde-3-phosphate dehydrogenase
PI3K: Phosphoinositide 3-kinases
IL12: Interleukin 12
AKT: Protein kinase B, also known as PKB
CXCL9: Chemokine (C-X-C motif) ligand 9
SOCS1: Suppressor of cytokine signaling 1
CYP2E1: Cytochrome P450 2E1
GWAS: Genome-wide association study
TRAPPC9: Trafficking protein particle complex 9
mTORC1: Cytosolic arginine sensor for mTORC1 subunit 1
JAK2: Janus kinase 2
STAT5A: Signal transducer and activator of transcription 5A
IDO2: Indoleamine 2,3-dioxygenase 2
NR4A1: Nuclear receptor subfamily 4 group A member 1
