# Supplementary figures and images for: Identification of novel molecular markers of mastitis caused by Staphylococcus aureus using gene expression profiling in two consecutive generations of Chinese Holstein dairy cattle
Source: J Anim Sci Biotechnol. 2020 Sep 28;11:98. doi: 10.1186/s40104-020-00494-7 (PMC7488426; doi:10.1186/s40104-020-00494-7)

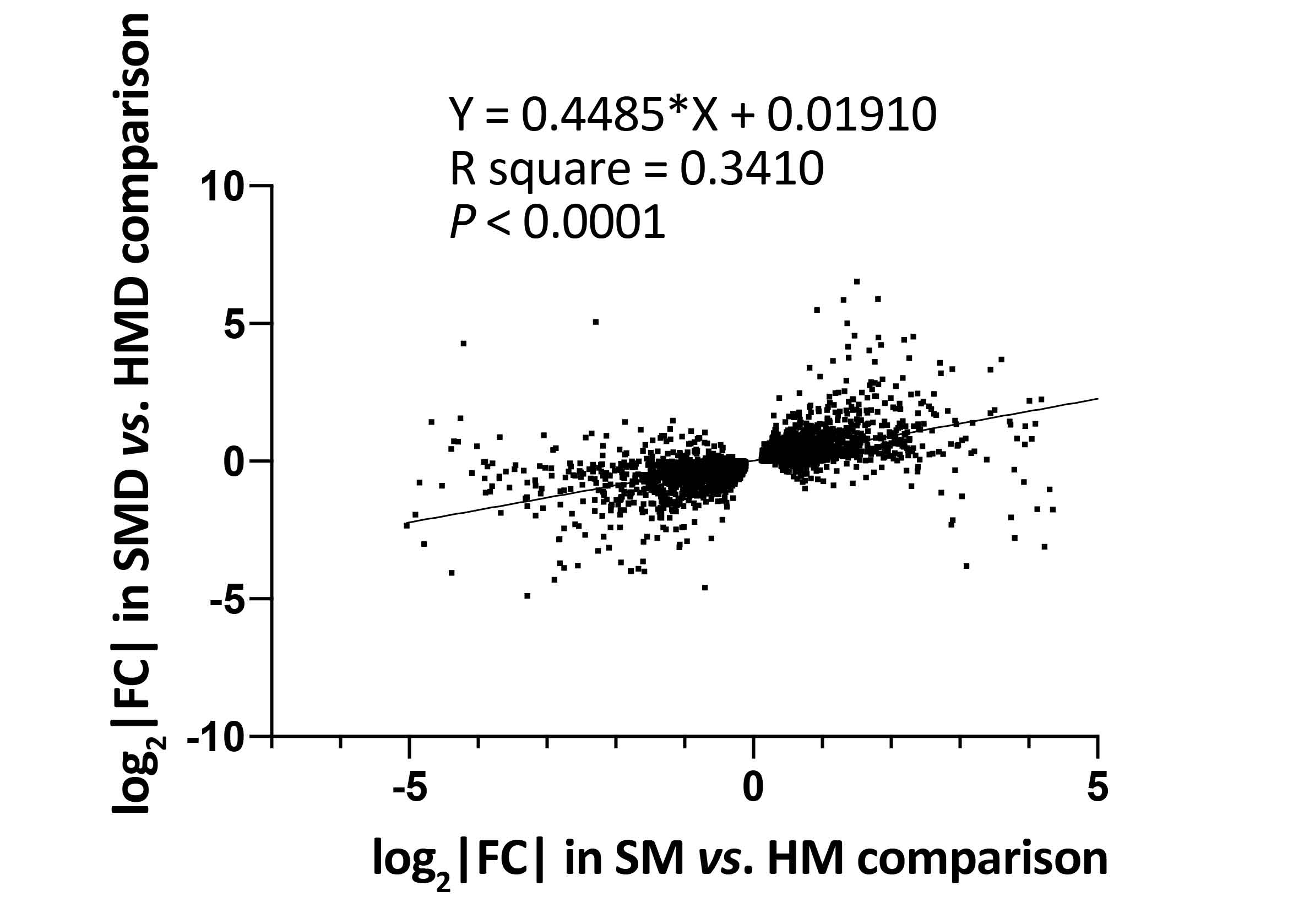

Supplement: Supplementary file 1 — Additional file 1: Supplementary Figure S1. Linear regression analysis of the log2|FC| of 4000 randomly selected gene expression values for the SM vs. HM and SMD vs. HMD comparisons. [file 40104_2020_494_MOESM1_ESM.jpg]

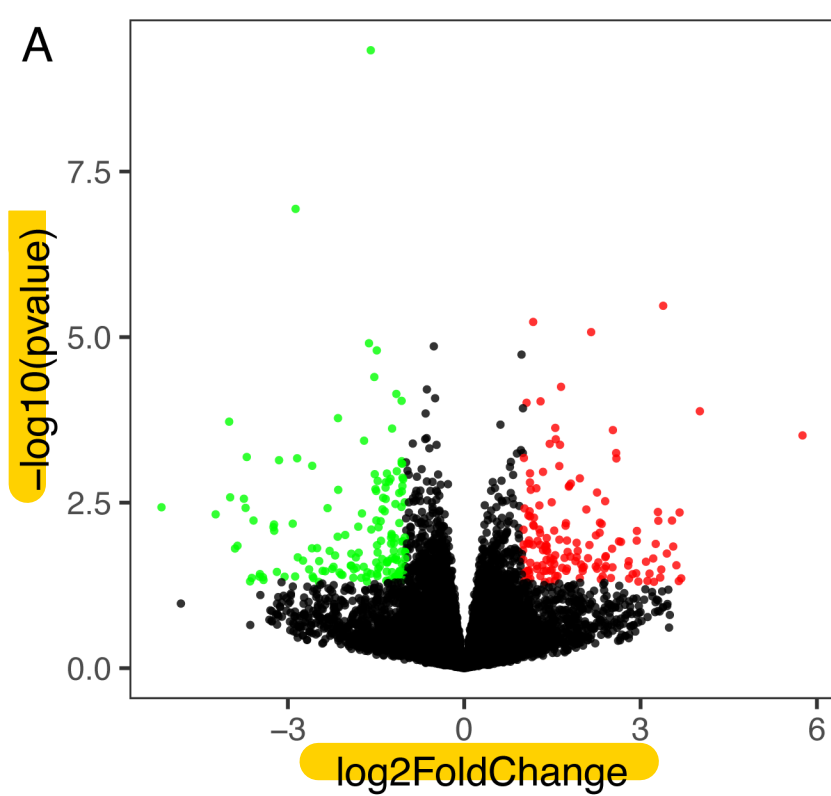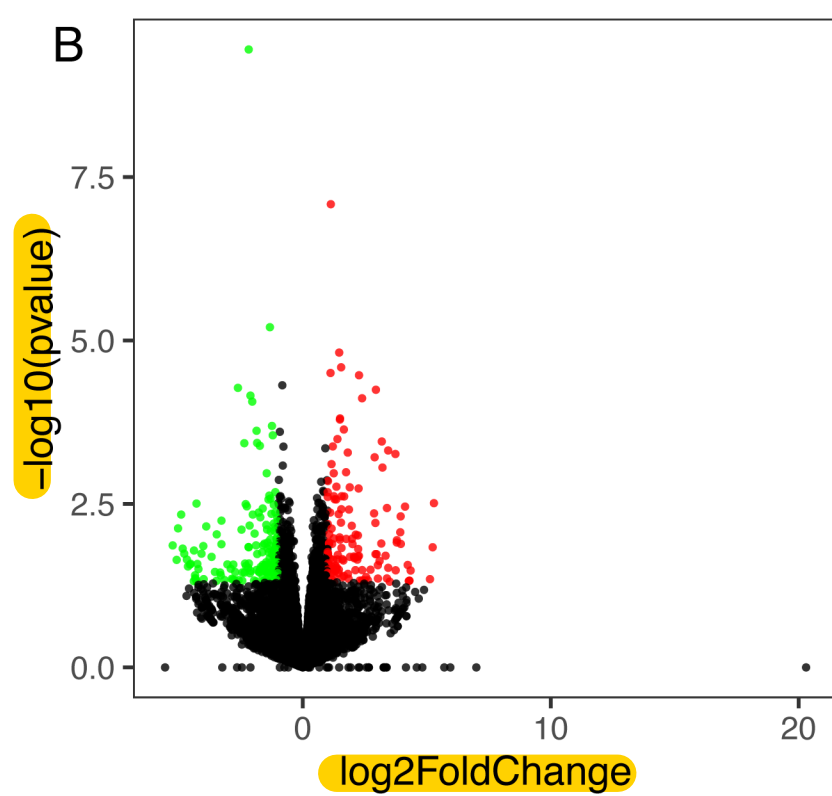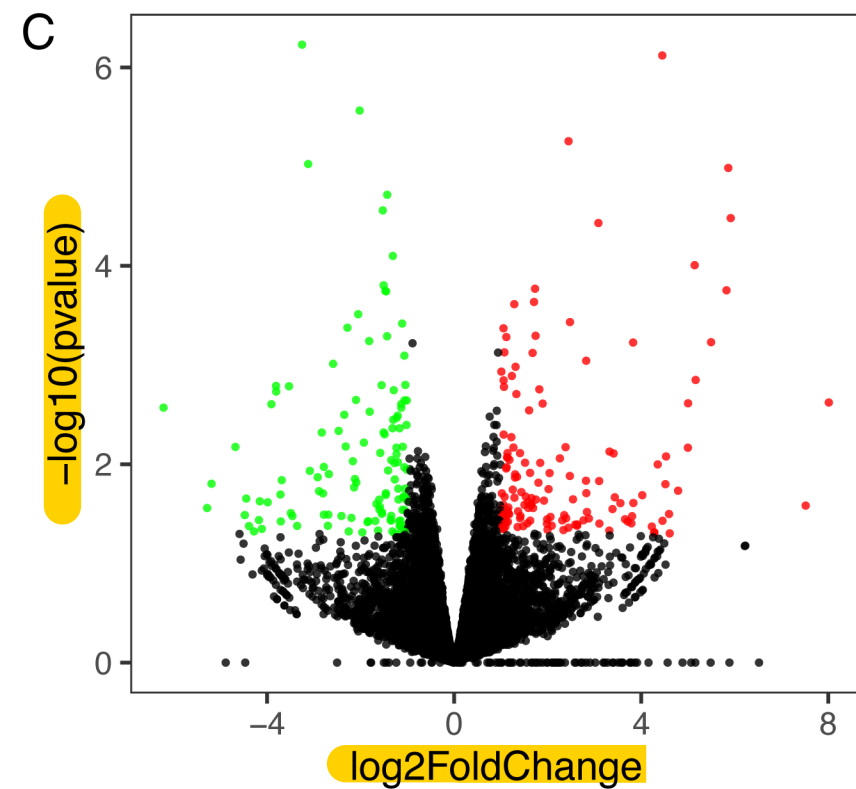

- Up-regulated
- Down-regulated
- Not significant

Supplement: Supplementary file 2 — Additional file 2: Supplementary Figur S2. Volcano plots of DEGs for (A) the S vs. H comparison, (B) the SM vs. HM comparison, and (C) the SMD vs. HMD comparison. [file 40104_2020_494_MOESM2_ESM.pdf]

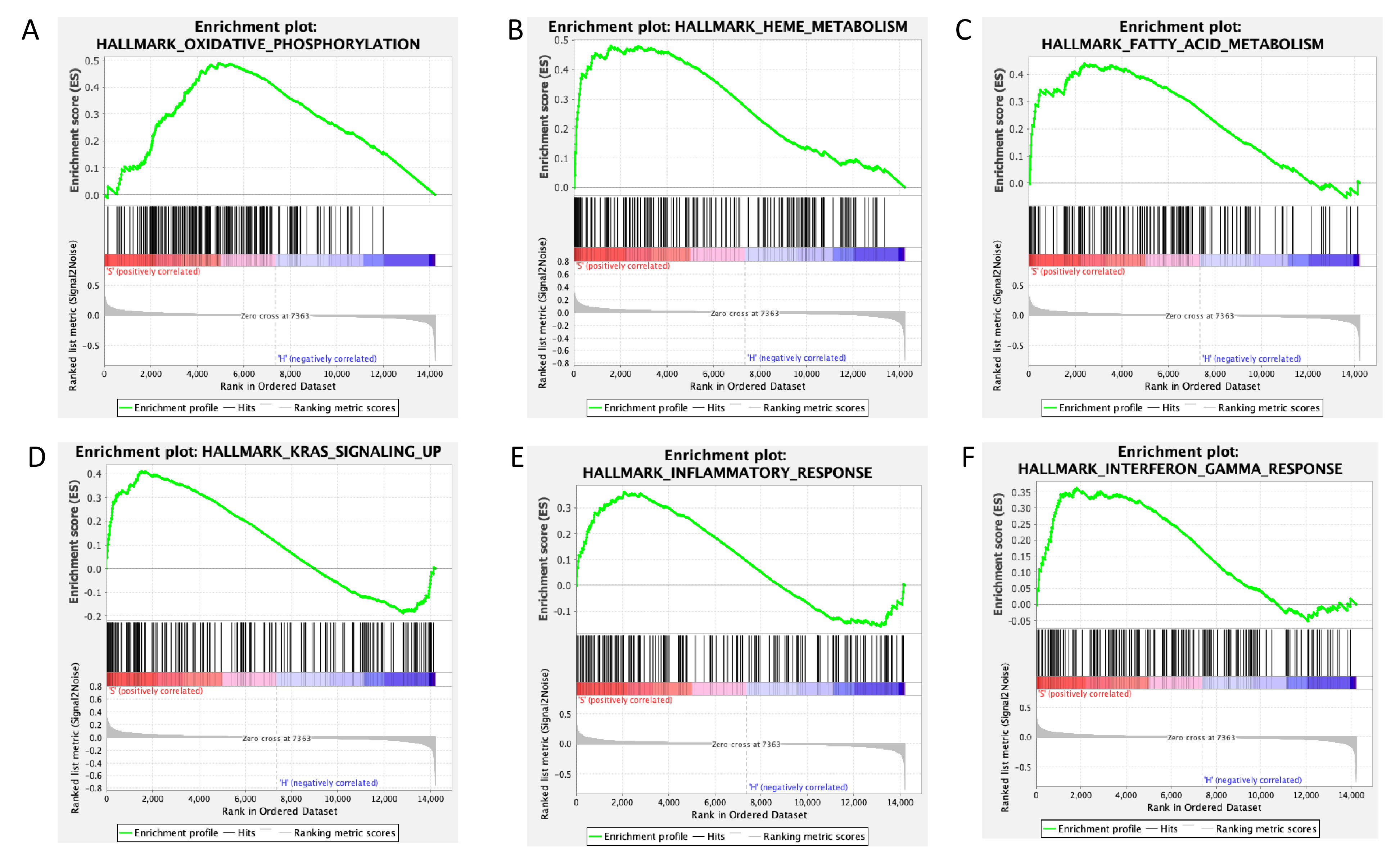

Supplement: Supplementary file 3 — Additional file 3: Supplementary Figure S3. Gene set enrichment analysis (GSEA) plots depicting the enrichment of functional gene sets up-regulated in the S group compared to the control group (FDR < 0.25 and P < 0.05). (A-F): GSEA plot depicting the enrichment of functional gene sets up-regulated in oxidative phosphorylation, heme metabolism, fatty acid metabolism, Kras signaling up-regulation, inflammatory response and interferon-gamma response in the S vs. H comparison (FDR < 0.25 and P < 0.05). [file 40104_2020_494_MOESM3_ESM.jpg]

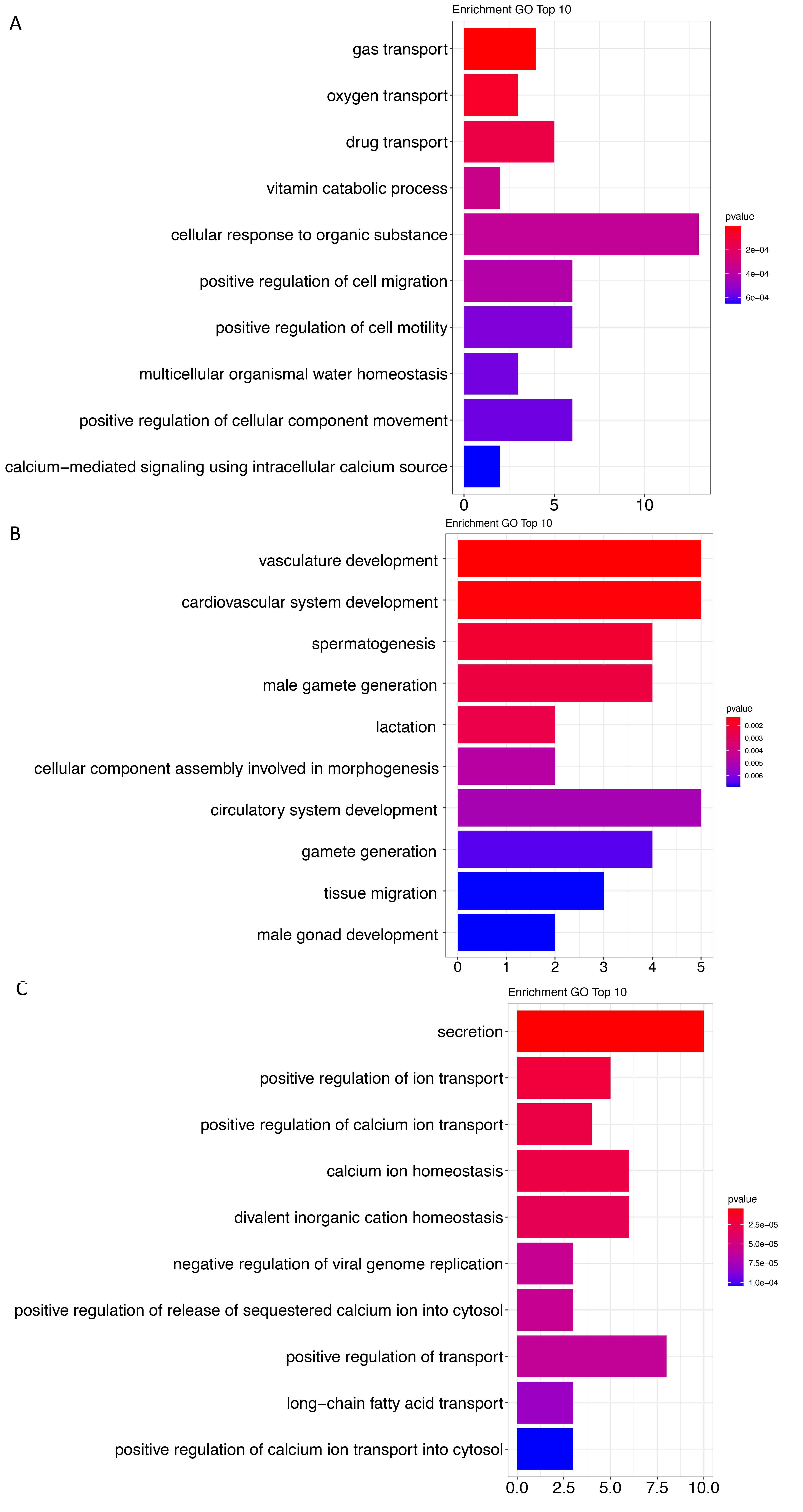

Supplement: Supplementary file 4 — Additional file 4: Supplementary Figure S4. GO Biological Process enrichment of DEGs. (A) Top ten Biological Process GO categories of DEGs in the S vs. H comparison. (B) Top ten Biological Process GO categories of DEGs in the SM vs. HM comparison. (C) Top ten Biological Process GO categories of DEGs in the SMD vs. HMD comparison. [file 40104_2020_494_MOESM4_ESM.jpg]
