# Supplementary material for: Identification of novel molecular markers of mastitis caused by Staphylococcus aureus using gene expression profiling in two consecutive generations of Chinese Holstein dairy cattle
Source: J Anim Sci Biotechnol. 2020 Sep 28;11:98. doi: 10.1186/s40104-020-00494-7 (PMC7488426; doi:10.1186/s40104-020-00494-7)
Supplement: Supplementary file 6 — Additional file 6: Table S2. Primer pairs of DEGs used for qRT-PCR validation. [file 40104_2020_494_MOESM6_ESM.docx]

**Table S2.** **Primer pairs of DEGs used for qRT-PCR validation.**

| Gene name | Primer sequence (5' to 3') |
| --- | --- |
| *ALAS2* | F: AGCCCTCAGATGATGGAAGA |
|  | R: CCCATGTTCCCAAAGTAGGA |
| *ACTA2* | F: TCCACGAGACCACGTACAAC |
|  | R: GCCACCAATCCAGACAGAGT |
| *RFX2* | F: ATGGTGATCCGAGACCTGAC |
|  | R: GAGGCCAGATCGTTGAACTC |
| *BLA-DQB* | F: GAGCAAGATGCTGAGTGGTG |
|  | R: CAGAAGAGCAAAACCAATCCCC |
| *MTUS1* | F: GCAATCTCGAGGCAACTTTC |
|  | R: CAGAGCAGCTCCTCGTTCTC |
| *C1R* | F: CAGGTGCAGGATCAAGGACT |
|  | R: CACCTTTGGTGTGCATCTTG |
| *CD24* | F: CTGGCGCTGCTCTTACCTAC |
|  | R: AGGGGCAGGTGAGGTAGTCT |
| *IDO1* | F: ATGTTCTCCTGGGCATTCAG |
|  | R: TGAGCTGGTGGCATGTATGT |
| *MX1* | F: GCATTGCAACAGGTCAGAGA |
|  | R: GCGGTCAGGTGTTGAAAGAT |
| *FCGR3A* | F: CTCTGAAGTGCCAGGGAGAC |
|  | R: TTGTACTCGCCACTGTCCTG |
| *ISG15* | F: GAGCGTGTACAAGCAGACCA |
|  | R: TTCATGAGGCCGTATTCCTC |
| *OAS1Y* | F: TTTGGTCTGGCTGGATTACC |
|  | R: TAGGCCTGGAACATCAGGTC |
| *PRKCI* | F: GCAGGTGGTACCTCCGTTTA |
|  | R: CATCAGGAGTGAGCTGGACA |
| *TAC3* | F: CTCCAAGTGTGGAATGAGCA |
|  | R: GCCAGTGGGACAGAGAGAAG |
| *SLC20A2* | F: GATCCTTCCGTTCGTGTGAT |
|  | R: GTGGACAGTGTGGTCTGGAA |
| *GATA2* | F: CCTGCGGCCTCTACTACAAG |
|  | R: ACACACCTGGACAGCTCCTC |
| *NRIP3* | F: AAGCAAGAGTCGGAGGAGTG |
|  | R: ACAGTCTTCCACGGAGATGG |
