# Supplementary material for: Identification of novel molecular markers of mastitis caused by Staphylococcus aureus using gene expression profiling in two consecutive generations of Chinese Holstein dairy cattle
Source: J Anim Sci Biotechnol. 2020 Sep 28;11:98. doi: 10.1186/s40104-020-00494-7 (PMC7488426; doi:10.1186/s40104-020-00494-7)
Supplement: Supplementary file 8 — Additional file 8: Table S4. Six significantly enriched upregulated gene sets. [file 40104_2020_494_MOESM8_ESM.docx]

| Table S4. Six significantly enriched upregulated gene sets | | | | |
| --- | --- | --- | --- | --- |
| Gene sets | Size | ES | P-value | FDR |
| Oxidative phosphorylation | 197 | 0.48850688 | 0 | 0.00165217 |
| Heme metabolism | 174 | 0.47988334 | 0 | 0.00516334 |
| Fatty acid metabolism | 140 | 0.44082037 | 0 | 0.03808289 |
| KRAS signaling up | 167 | 0.41130528 | 0 | 0.06784321 |
| Inflammatory response | 172 | 0.36158007 | 0.007142857 | 0.24806002 |
| Interferon gamma response | 176 | 0.36299467 | 0.017456358 | 0.22911716 |

Note: Size: number of genes contributing to the leading-edge subset within the gene set (number of genes in the gene set after filtering out those genes not in the expression dataset); ES: enrichment score; *P*-value: normal *P*- value not adjusted for gene size or multiple testing; a *P*-value of zero (0) indicates an actual *P*-value of less than 0.001; FDR: False discovery rate.
